# Antimicrobial Activity of Oleanolic and Ursolic Acids: An Update

**DOI:** 10.1155/2015/620472

**Published:** 2015-02-22

**Authors:** Jéssica A. Jesus, João Henrique G. Lago, Márcia D. Laurenti, Eduardo S. Yamamoto, Luiz Felipe D. Passero

**Affiliations:** ^1^Laboratório de Patologia de Moléstias Infecciosas, Departamento de Patologia, Faculdade de Medicina da Universidade de São Paulo, Avenue Dr. Arnaldo 455, 06780-210 Cerqueira César, SP, Brazil; ^2^Instituto de Ciências Ambientais, Químicas e Farmacêuticas, Universidade Federal de São Paulo, Rua Professor Artur Riedel 275, 09972-270 Diadema, SP, Brazil

## Abstract

Triterpenoids are the most representative group of phytochemicals, as they comprise more than 20,000 recognized molecules. These compounds are biosynthesized in plants via squalene cyclization, a C_30_ hydrocarbon that is considered to be the precursor of all steroids. Due to their low hydrophilicity, triterpenes were considered to be inactive for a long period of time; however, evidence regarding their wide range of pharmacological activities is emerging, and elegant studies have highlighted these activities. Several triterpenic skeletons have been described, including some that have presented with pentacyclic features, such as oleanolic and ursolic acids. These compounds have displayed incontestable biological activity, such as antibacterial, antiviral, and antiprotozoal effects, which were not included in a single review until now. Thus, the present review investigates the potential use of these triterpenes against human pathogens, including their mechanisms of action, via *in vivo* studies, and the future perspectives about the use of compounds for human or even animal health are also discussed.

## 1. Introduction

The triterpenoids are the most representative group of phytochemicals; they comprise more than 20,000 recognized compounds and are biosynthesized in plants through squalene cyclization [1]. The triterpenes can be classified into groups based on their structural skeletons: cucurbitanes, cycloartanes, dammaranes, euphanes, friedelanes, holostanes, hopanes, isomalabaricanes, lanostanes, lupanes, oleananes, protostanes, tirucallanes, and ursanes, among others [2].

The diversity of triterpenes is highly associated with their broad range of pharmacological effects. In Asian countries, triterpenes are traditionally used as anti-inflammatory, analgesic, hepatoprotective, cardiotonic, and sedative agents [3]. Other studies have also demonstrated their antioxidant, antiallergic, antipruritic, antiangiogenic, and antimicrobial potential [4]. In addition, some studies have already demonstrated that several of these compounds exhibit anticancer potential, with high selectivity for cancer cells and the ability to induce apoptosis-related death in most cases [5–10]. Due to this specific action, several triterpenoids are currently being evaluated in phase I clinical trials [11].

**Figure 1:**
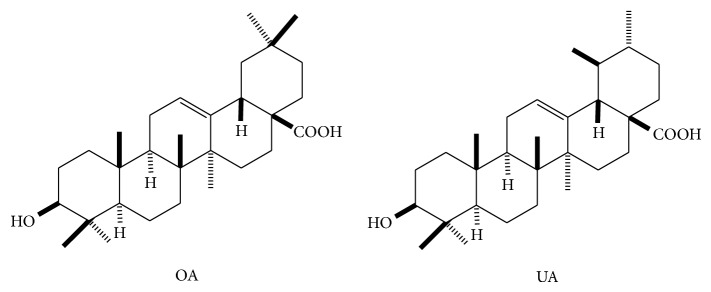
Skeleton of oleanolic acid (OA) and ursolic acid (UA).

Oleanolic acid (OA) and its isomer, ursolic acid (UA), are triterpenoid compounds that widely occur in nature in free acid form or as an aglycone precursor for triterpenoid saponins [12]. These triterpenoid acids frequently occur simultaneously because they share similar structural features. These compounds have also shown similar pharmacological activities, such as hepatoprotective, anti-inflammatory, antioxidant, and anticancer effects, which may be attributable to the different substructures in A, C, and E rings or other positions (Figure 1).

## 2. Oleanolic Acid (OA)

OA (3*β*-hydroxyolean-12-en-28-oic acid) is a pentacyclic triterpenoid with widespread occurrence throughout the plant kingdom. This compound and its derivatives possess several interesting pharmacological activities, such as anti-inflammatory, antioxidant, anticancer, and hepatoprotective effects. OA was previously isolated from almost 2000 plant species [12–14], and the main source of this compound includes plants belonging to the Oleaceae family, such as* Olea europaea* (the olive) [15, 16]. In plants, the biological roles of this compound are often associated with the formation of a barrier against water loss and pathogens [17]. Moreover, allelopathic properties have already been described for this compound [18]. Several medicinal plants produce and accumulate OA and its derivatives as their main metabolites, which could be directly associated with their biological activities, as shown in Table 1.

## 3. Ursolic Acid (UA)

UA (3*β*-hydroxyurs-12-en-28-oic acid) is a pentacyclic triterpenoid compound that shares a common cooccurrence with OA in several plant species; however, it features a more restricted distribution when compared to OA [12, 84]. This compound has been found in large amounts in berries (such as cranberries) and mostly in the peel [85]. Similar to what is observed with OA, the biological role of UA in plants seems to be associated with protection against herbivores and pathogens [86]. The occurrence of UA and its derivatives as major metabolites in medicinal plants could be associated with their biological activities, as shown in Table 2.

Many comprehensive reviews of OA and UA have been published and have covered different areas of interest, such as their isolation, structural determination, and pharmacological activities [12, 87–90].

In spite of the pharmacological effects that have already been demonstrated, different reports have shown that OA and UA exhibit antimycotic, antitumoral, antibacterial, antiviral, and antiparasitic properties [4, 9, 26, 91–95], suggesting that these compounds are important classes of prototypical natural antibiotic molecules. This review aims to summarize the information regarding the microbiocidal activities of both OA and UA, highlighting the importance of these compounds as leading molecules with pharmacological and medical importance in the development of new drugs.

## 4. Microbicidal Effects of Oleanolic and Ursolic Acids

### 4.1. Antibacterial Properties of Oleanolic and Ursolic Acids

The antibacterial properties of OA and UA were assayed against different bacterial species, and the obtained results suggested the importance of these compounds as antibiotic drugs.

One of the first studies that aimed to evaluate the possible effect of OA and UA against bacteria was developed by Kozai et al. [96]. In this work, it was demonstrated that both of these triterpenes inhibited the synthesis of insoluble glucan, catalyzed by crude glucosyltransferase (GTase) from cariogenic* Streptococcus mutans*. Recently, the potential of UA against* S. mutans* and* S. sobrinus* was reinforced with a minimum inhibitory concentration (MIC)_50_ of 2.0 *μ*g/mL [97], indicating that these compounds can inhibit caries in teeth.

When used against* Mycobacterium tuberculosis*, which is a bacterium that affects around one-third of the human population and represents the infection that causes the most deaths worldwide, it was found that OA isolated from* Lantana hispida* was also effective at displaying a MIC value of 25 *μ*g/mL [49]. In addition, a MIC of 50 *μ*g/mL was reported when OA was used against* M. tuberculosis* streptomycin-, isoniazid-, rifampin-, and ethambutol-resistant strains. Similar to OA, UA purified from* Chamaedorea tepejilote* leaves was capable of eliminating* M. tuberculosis* at 100 *μ*g/mL [98], suggesting that there is a potential for both compounds to kill this pathogen.

The diversity of the antibacterial properties of OA and UA has also been illustrated against other human bacterial pathogens, such as* S. pneumonia* (MIC of 16 *μ*g/mL), methicillin-sensitive and methicillin-resistant* Staphylococcus aureus* (MIC of 8 *μ*g/mL and 64 *μ*g/mL, resp.) [129],* Bacillus subtilis* (MIC of 8 *μ*g/mL),* B. cereus*,* Enterococcus faecalis* (MIC of 6.25–8.00 *μ*g/mL),* E. faecium* (MIC of 8 *μ*g/mL), and* Pseudomonas aeruginosa* (MIC of 256 *μ*g/mL) [130–132].

Although few works have examined the mode of action of these triterpenes, studies conducted with* E. coli* demonstrated that OA can moderately affect the efflux of pumps, which could directly interfere with the viability of this species [133]. Other mechanisms of action of OA can be associated with the induction of a stress response. Grudniak et al. [134] observed that* E. coli* treated with OA altered the synthesis of DnaK, thus inducing the heat-shock response in this species. Kurek et al. [135] also verified that both OA and UA inhibited peptidoglycan turnover in* Listeria monocytogenes*, affecting the amount of muropeptides and, ultimately, the cellular wall of bacteria, suggesting that this biochemical pathway can be a target for both triterpenes.

Taken together, these works suggest that OA and UA possess a broad range of antibacterial activity, mainly against gram-positive bacteria. In addition, all of these works have alerted us to the important classes of prototype drugs that can be derived from these triterpenes, including the development of drugs that can be used against infections caused by drug-resistant bacteria species.

### 4.2. Antiviral Properties of Oleanolic and Ursolic Acids

The antiviral properties of OA and UA have been studied since the 1990s, specifically those used against human immunodeficiency virus (HIV) and the hepatitis virus. HIV belongs to the Retroviridae family and the genus,* Lentivirus*, which produces characteristically slow and progressive infection [136]. One of the first works [137] dealing with this subject showed that UA purified from* Cynomorium songaricum* (Cynomoriaceae) inhibited HIV-1 protease in a dose-dependent manner (inhibitory concentration [IC]_50_ of 8 *μ*g/mL). OA and its derivatives were also capable of inhibiting HIV-1 protease, with an IC_50_ of 4–20 *μ*g/mL [138]. The inhibition of this enzyme produces immature and noninfectious virions and molecules, consequently blocking the life cycle of HIV [139]; this will ultimately improve the patient's quality of life. In addition,* ex vivo* experiments showed that peripheral blood mononuclear cells (PBMC) from HIV-infected patients, which were incubated with different doses of OA, presented significant reduction of viral replication, which was comparable with the drug, azidothymidine (AZT). Similar results were found when PBMC from healthy donors were infected with HIV-1, yielding an effective concentration (EC)_50_ of 22.7 *μ*M and 24.6 *μ*M, respectively [140]. Moreover, [141] demonstrated that OA was capable of eliminating, with high selectivity, HIV (therapeutic index [TI] ratio of 12.8) when compared to the H9 cell lineage; however, the AZT drug presented with the highest TI, which was 41.667.

The potential of OA and UA was also determined against hepatitis B and C viruses (HBV and HCV, resp.). These viruses are of serious concern for human populations, since approximately 500 million people are chronically infected with one or both viruses, resulting in fibrosis and cirrhosis of the liver, and ultimately leading to the development of hepatocellular carcinoma [142, 143]. Although vaccines and therapeutic strategies against these viruses already exist, new drug prototypes are under development, such as OA and UA. In this regard, it was demonstrated that UA primarily decreased the migratory process and matrix metalloproteinase-3 secretion in HBV X protein-transactivated cell lineages. In addition, UA-treated cells were more sensitive to transforming growth factor- (TGF-) *β*-mediated apoptosis than were the control cells.* In vivo* experiments showed that HBV-induced tumors were significantly lower in UA-treated animals when compared to controls [144]. These interesting studies showed that UA could block the pathological effects of HBV in cell lineages, suggesting that new classes of antiviral drugs could be developed using UA. In contrast, OA isolated from* Ligustri lucidi* seems to be very effective at eliminating intracellular HCV with an IC_50_ of 5.5 *μ*g/mL and a high selectivity index (SI) of 30.8. Otherwise, the IC_50_ found for UA activity was higher than that determined for OA (IC_50_ of 33.8 *μ*g/mL), and the latter featured a lower SI (6.7). In addition, one possible mechanism of action of OA was related to the suppression of the viral NS5B RdRp enzyme, which is a central enzyme responsible for HCV RNA replication [145].

UA and OA were also assayed against the proliferation of herpes viruses in host cells. Herpes simplex viruses (HSV) cause herpes labiles, herpes genitalis, keratitis, and encephalitis. The HSV infection caused by type-1 and type-2 viruses is mainly transmitted through close personal contact. The therapy that is used against the infection has severe side effects, and drug-resistant viruses have been detected [146], justifying the rationale to search for new drugs. In this regard, ethnomedicinal studies conducted in India showed that some plants used to treat skin problems, such as* Mallotus peltatus* and* Achyranthes aspera* [147, 148], produce appreciable amounts of UA and OA [149]. Considering that herpes infections affect the skin and mucosa, Bag et al. [146] and Mukherjee et al. [150] assayed crude extracts of, and active fractions derived from,* M. peltatus* and* A. aspera*, which contained UA and OA. The researchers found that both fractions presented with strong inhibitory activity against HSV-1 and HSV-2, which was comparable to the standard drug, Aciclovir. In addition, the OA-containing fraction from* A. aspera* triggered interleukin- (IL-) 12 production in treated peritoneal macrophages [150], which is an important cytokine that is responsible for activating the CD4^+^Th1 cell population and for eliminating intracellular pathogens [151, 152].

These works indicate that OA and UA inhibit viral spreading in different host cell lineages with high levels of sensitivity and selectivity; this mainly depends upon the virus type and the host cell. In addition, the mechanism of action of both triterpenes was related to the control of virus replication and also to the immunomodulatory effect on the host cells, suggesting that new drugs can be developed from these structures.

### 4.3. The Antiprotozoal Properties of OA and UA

OA and UA also displayed appreciable antiparasitic effects against* Plasmodium falciparum*,* Toxoplasma gondii*,* Trypanosoma cruzi*, and* Leishmania* sp.

The parasitic disease with the greatest impact is malaria; it affects around 40% of the world's population, spanning across more than 100 countries, and its etiological agent is a protozoa belonging to the genus,* Plasmodium* [153]. Although different drugs can eliminate this parasite, the problem with the* Plasmodium* sp. is that its resistance needs to be overcome [154]; this indicates that the search for new antimalarial compounds is necessary and urgent.

In this regard, one of the first works to demonstrate the antimalarial properties of triterpenes against chloroquine-resistant and chloroquine-sensitive* Plasmodium falciparum* was conducted by Steele et al. [155]. In this study, OA and UA were purified from ethanolic extract, which was prepared from the root barks of* Uapaca nitida* (Euphorbiaceae). UA showed antimalarial effects with an IC_50_ of 36.5 *μ*g/mL and 28 *μ*g/mL against chloroquine-resistant and chloroquine-sensitive strains, respectively. Otherwise, the IC_50_ that was found for OA was 88.8 *μ*g/mL and 70.6 *μ*g/mL for chloroquine-resistant and chloroquine-sensitive strains, respectively. Other studies have also corroborated the potential of UA, purified from* Mitragyna inermis*, against chloroquine-sensitive and chloroquine-resistant strains, showing an IC_50_ between 15 *μ*g/mL and 18 *μ*g/mL. In addition, infected blood cells treated with UA presented with lower parasitism than did infected controls [94]. Other studies have also demonstrated that OA and UA purified from* Satureia parvifolia*,* Mimusops caffra*,* M. obtusifolia*, and* Kleinia odora* were able to eliminate* P. falciparum* [156–158].

Drugs based on pentavalent antimonials, Amphotericin B, nifurtimox, and benznidazole, are employed to treat patients with leishmaniasis and American trypanosomiasis but, unfortunately, these drugs are toxic and reports of parasite resistance to them have been constantly published, justifying the search for new active compounds. In infections caused by trypanosomatids, OA and UA were also tested, first in the use against* Leishmania* sp. parasites, and then against* Trypanosoma cruzi*, the etiological agents of leishmaniasis and American trypanosomiasis, respectively. Leishmaniasis is a complex disease, and its symptoms range from the presence of severe cutaneous lesions to the more visceral form of the disease, which affects the spleen, liver, and bone marrow [159].

Tan et al. [160] evaluated the leishmanicidal potential of OA and UA extracted from* Salvia cilicica* roots. The obtained results showed that UA was primarily active against intracellular amastigote forms of* L. donovani* and* L. major*, with an IC_50_ of 12.7 nM and 7.0 nM, respectively. These values were comparable to the standard drug, Pentostam, whose IC_50_ was 10.6 nM and 9.8 nM against the same parasite species, respectively.* L. (L.) amazonensis* promastigotes were shown to be highly sensitive to OA and UA, presenting an IC_50_ of 10 *μ*g/mL and 5 *μ*g/mL, respectively. In addition, both of these compounds were active against the intracellular form of* L. (L.) amazonensis*, showing an IC_50_ of 27 *μ*g/mL and 11 *μ*g/mL, respectively. On the other hand, an IC_50_ of 83 *μ*g/mL was obtained for experimental treatment with meglumine antimoniate [95], suggesting that these triterpenes are more effective than one of the standard drugs that is currently used to treat patients. The effect of these triterpenes on amastigote forms was not related to nitric oxide production, since elevation of this effector molecule was not verified in infected macrophages. Further studies also demonstrated that UA was active against promastigote forms of* L. (L.) amazonensis*,* L. (L.) infantum* [161], and* L. (L.) donovani* [162].

Recently, a bioguided study conducted with extracts of* Baccharis uncinella* leaves led to the identification of a bioactive fraction that contained OA and UA triterpenes. This fraction showed moderated activity against* L. (V.) braziliensis* and* L. (L.) amazonensis* promastigotes, although it was very active against amastigote forms of both parasite species; moreover, the leishmanicidal effect could be related to a direct effect on the amastigote forms. Additionally, these compounds triggered nitric oxide production in the macrophages, since infected cells incubated with the highest concentration of this fraction produced significant amounts of this effector molecule [26]. Due to this leishmanicidal potential, this fraction (OA + UA) was assayed as a prototype drug in* L. (L.) amazonensis*-infected mice. Animals that were treated with 1.0 mg/kg and 5.0 mg/kg of triterpene fraction presented with reduced lesion sizes and skin parasitism, which was accompanied by a significant elevation of IL-12 and interferon- (IFN-) *γ* cytokines. Furthermore, the treatment did not alter the histological profile of the spleen, liver, heart, lungs, and kidneys of mice [27]. Interestingly, a total dose of 1.25 mg of amphotericin B was required to eliminate 86% of parasites, while only 0.625 mg of the triterpene fraction was required to inhibit approximately 93% of skin parasitism, suggesting the elevated leishmanicidal potential of OA and UA.

**Figure 2:**
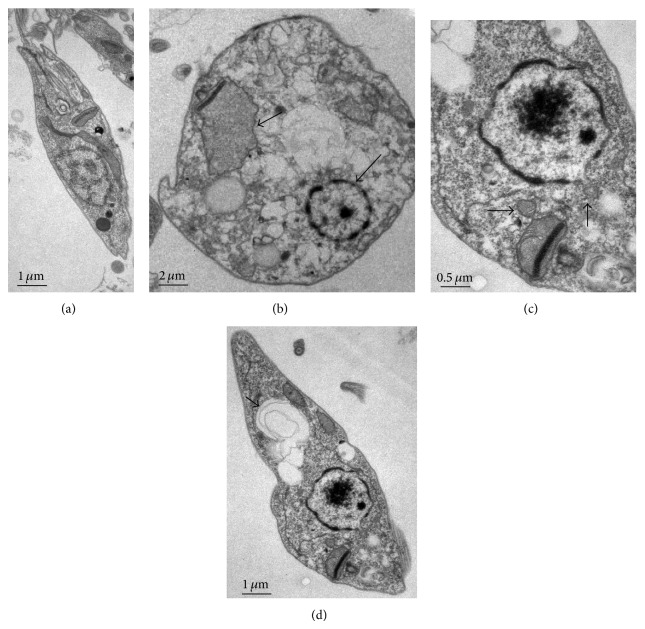
Ultrastructural alterations induced by 10.96 *μ*g of UA on promastigote forms of* L. (L.) amazonensis*. (a) Control parasites showed a normal morphology of the cell membranes, nucleus, and kinetoplast (20.000x). (b) Parasites treated with UA presented with evident external and internal alterations, such as mitochondrial swelling (arrowhead) and a pyknotic nucleus (short arrow) (10.000x); (c) Blebs (arrows) were detected in the nucleus and kinetoplast (40.000x); and (d) membranes were detected inside vacuoles, as indicated by the arrow (20.000x).

In addition, our group demonstrated, through ultrastructural studies, that* L. (L.) amazonensis* promastigote forms treated with 10.96 *μ*g of UA presented with irreversible morphological changes after 18 hours of incubation. Control parasites presented with normal membrane morphology, cytoplasm, nucleus, mitochondrion, and flagellum (Figure 2(a)). Otherwise, treated parasites presented with rounded-shape morphology, and the intracellular environment presented with vacuoles, suggesting organelle degradation (Figure 2(b)) and swelling of the mitochondrion, and a pyknotic nucleus was detected (Figure 2(b)); blebs were also visualized in the nucleus and in the kinetoplast (Figure 2(c)). In addition, intracellular vacuoles presented with fragments of membranes (Figure 2(d)), suggesting degradation of the organelles. Taken together, these results suggest that, in promastigote forms of* L. (L.) amazonensis*, UA induces a mechanism of death associated to apoptosis or even autophagy. This is the first study that depicted the possible mechanism of action of UA on* L. (L.) amazonensis* promastigote forms.

Based on previous works, these triterpenes can be regarded as antileishmanial agents since these studies demonstrated that these agents can be more effective than conventional drugs. In addition, more attention needs to be paid to UA, which is the primary antileishmanial agent when compared to its isomeric derivative, OA.

In American trypanosomiasis, the parasite* T. cruzi* infects a broad range of cell types, preferentially, muscle cells from the gut and heart, leading to a loss of organ function [163, 164]. Unfortunately, there are only two drugs that can be used to treat patients (nifurtimox and benznidazole), which are associated with serious side effects and are effective only in the acute phase of the disease [165], indicating that a search for a new trypanocidal compound is necessary. OA and UA purified from* Miconia* species were shown to be active against the blood form of* T. cruzi*; they showed an IC_50_ of 80.4 *μ*M and 21.3 *μ*M, respectively, while the IC_50_ for gentamicin violet was 71.6 *μ*M [92], reinforcing the antiparasitic potential of UA. These interesting results led to the evaluation of the therapeutic potential of OA and UA triterpenes in a murine model of American trypanosomiasis. Animals treated with 2.0 mg/kg of OA, UA, and a mixture of OA plus UA presented with low parasitemia when compared to animals treated with benznidazole [166]. Ferreira et al. [167] also demonstrated that OA and UA were capable of controlling the peak of parasitemia in infected mice and, interestingly, treated mice did not show any alterations in their biochemical parameters, reinforcing the idea that these triterpenes are not toxic for animals. Considering the low or absent level of toxicity of triterpenes for mice, as well as their high trypanocidal activity, these results suggest that both compounds can be used for the development of new drugs against* T. cruzi.*

## 5. Conclusion

Several triterpenes, which displayed interesting structural features, have been considered inactive for a long period of time. However, different works have since demonstrated the wide array of pharmacological activities inherent in this class of natural compounds.

Specifically, UA and OA present remarkable antimicrobial activities, and they act against important human pathogens such as mycobacteria, HIV, and different protozoal species. The present review described interesting works about the antimicrobial action of UA and OA that, in fact, could be considered drug prototypes. In spite of this, the present review also alerted us to some concerns, insofar as the majority of the works presented here have not depicted the possible mechanism of action of these triterpenoids in microorganisms. Moreover, studies have not associated the* in vitro* potency of these agents with studies dealing with their therapeutic action (*in vivo*); this should be a priority in this field. In addition, these types of strategies will be crucial in the development of new drugs that can be used for populations that are at risk for contracting certain diseases.

## Figures and Tables

**Table 1:** Oleanolic acid's (OA) derivatives and their biological activities.

Plant species (family)	Biological activity	Reference
*Aceriphyllum rossii* (Saxifragaceae)	Cytotoxic	[19]
	Anticomplement activity	[20]
		[21]

*Actinidia chinensis* (Actinidiaceae)	Hepatoprotection	[22]

*Aralia chinensis* (Araliaceae)	Hepatoprotection	[23, 24]

*Astilbe chinensis* (Saxifragaceae)	Cytotoxic	[25]

*Baccharis uncinella* (Asteraceae)	Antileishmanial	[26]
		[27]

*Baeckea gunniana* (Myrtaceae)	Inhibition of *β*-DNA polymerase	[28]

*Beta vulgaris* (Chenopodiaceae)	Hepatoprotection	[29, 30]

*Betula ermanii* (Betulaceae)	Antitumor	[31]

*Calendula officinalis* (Compositae)	Antifungal activity	[32]

*Chrysosplenium carnosum* (Saxifragaceae)	Cytotoxic	[33]

*Diospyros kaki* (Ebenaceae)	Inhibition of tyrosine phosphatase	[34]

*Dysoxylum hainanense* (Meliaceae)	Antibacterial	[35]

*Eclipta prostrata* (Asteraceae)	Antifibrotic activity	[36]

*Embelia schimperi* (Myrsinaceae)	Antibacterial	[37]

*Eugenia jambolana* (Myrtaceae)	Inhibition of lipid peroxidation and protection against adriamycin toxicity; antifertility activity	[38–40]

*Fagus hayatae* (Fagaceae)	*α*-glucosidase inhibition	[41]

*Fatsia polycarpa* (Araliaceae)	Cytotoxic, antihepatitis B virus (HBV), and antibacterial	[42]

*Ganoderma lucidum* (Labiatae)	Anticariogenic activity	[43]

*Glechoma hederacea* (Labiatae)	Inhibition of azoxymethane-induced carcinogenesis in rats; Antitumor promotion	[44–46]

*Ilex kudincha* (Aquifoliaceae)	Inhibition of acyl CoA cholesteryl acyl transferase	[47]

*Junellia aspera* (Verbenaceae)	Cytotoxic	[48]

*Lantana hispida* Verbenaceae)	Antimycobacterial	[49]

*Liquidambar formosana* (Altingiaceae)	Inhibition of NFAT transcription factor	[50]

*Ligustrum lucidum* (Oleaceae)	Anti-inflammatory; antihyperglycemic; inhibition of mutagenicity by B(a)P	[51–54]

*Luffa cylindrica * (Cucurbitaceae)	Anti-inflammatory and inhibition of C3-convertase of the complement pathway	[55, 56]

*Lysimachia heterogenea* (Primulaceae)	Cytotoxic	[57]

*Lysimachia parvifolia* (Primulaceae)	Cytotoxic	[58]

*Nardophyllum bryoides* (Asteraceae)	Cytotoxic	[59]

*Microtropis japonica* (Celastraceae)	Cytotoxic	[60]

*Nigella glandulifera* (Ranunculaceae)	Cytotoxic	[61]

*Oleandra neriifolia* (Araliaceae)* *	Anti-inflammatory	[62]

*Panax ginseng* (Araliaceae)	Hepatoprotection	[63]

*Panax stipuleanatus* (Araliaceae)	Anticancer	[64, 65]
	Inhibition of NF-*κ*B	

*Phyllanthus flexuosus* (Euphorbiaceae)	Inhibition of DNA topoisomerases I and II	[66]

*Platycodon grandiflorum* (Campanulaceae)	Antiproliferative	[67]

*Rosa laevigata* (Rosaceae)	Anti-inflammatory	[68, 69]
	NF-*κ*B transcriptional activity	

*Sapindus mukorossi* (Sapindaceae)	Anti-inflammatory	[70]

*Siphonodon celastrineus* (Celastraceae)	Cytotoxic	[71, 72]

*Swertia mileensis* (Gentianaceae)	Hepatoprotection	[73–75]

*Swertia japonica * (Gentianaceae)	Hepatoprotection	[76]

*Terminalia arjuna* (Combretaceae)	Cardioprotection	[77]

*Terminalia chebula* (Combretaceae)	Cytotoxic	[78]

*Tetrapanax papyriferum* (Araliaceae)	Hepatoprotection	[79]

*Tinospora sagittata* (Menispermaceae)	Antihyperglycemic	[80]

*Uncaria laevigata* (Rubiaceae)	Inhibition of *α*-glucosidase	[81]

*Uncaria sessilifructus* (Rubiaceae)	Inhibition of activities against LPS-induced nitric oxide production in RAW264.7 macrophages	[82]

*Viburnum chingii* (Adoxaceae)	Cytotoxic	[83]

**Table 2:** Ursolic acid's (UA) derivatives and their biological activities.

Plant species (family)	Biological activity	Reference
*Actinidia chinensis* (Actinidiaceae)	Hepatoprotective	[99]

*Baeckea gunniana* (Myrtaceae)	Inhibition of *β*-DNA polymerase	[28]

*Callana vulgaris* (Ericaceae)	Inhibition of lipoxygenase and cyclooxygenase in HL-60 leukemic cells	[100, 101]

*Centella asiatica* (Mackinlayaceae)	Inhibition of NO	[102]

*Emmenopterys henryi* (Rubiaceae)	Cytotoxic	[103]

*Eribotrya japonica* (Rosaceae)	Inhibition of mutagenesis in bacteria	[104]

*Eucalyptus hybrid* (Myrtaceae)	Hepatoprotection	[105]

*Eucalyptus loxophleba* (Myrtaceae)	Antileishmanial	[106]

*Fragaria ananassa* (Rosaceae)	Cytotoxic	[107]

*Gentiana aristata* (Gentianacea)	Cytotoxic	[108]

*Glechoma hederacea* (Labiatae)	Antitumor promotion	[46]

*Ilex cornuta* (Aquifoliaceae)	Cytotoxic	[109]

*Leonurus cardiaca* (Lamiaceae)	Anti-inflammatory	[110]

*Melaleuca leucadendron* (Myrtaceae)	Inhibition of histamine release	[111]

*Microtropis japonica* (Celastraceae)	Cytotoxic	[60]

*Mulgedium tataricum* (Asteraceae)	Cytotoxic/antibacterial	[112]

*Nauclea officinalis* (Rubiaceae)	Inhibition of NO production	[113]

*Nardophyllum bryoides* (Asteraceae)	Cytotoxic	[59]

*Ocimum sanctum* (Labiatae)	Inhibition of lipid peroxidation and protection against adriamycin toxicity	[38, 39]

*Petasites tricholobus* (Asteraceae)	Antibacterial	[114]

*Potentilla fulgens* (Rosaceae)	Antioxidant	[115]

*Pyrola rotundifolia* (Pyrolaceae)	Anti-inflammatory	[116]

*Psychotria serpens* (Rubiaceae)	Cytotoxic to leukemia cells	[117]

*Rhododendron brachycarpum* (Ericaceae)	Inhibition of PTP1B	[118]

*Rosa laevigata* (Rosaceae)	Anti-inflammatory	[68]

*Rosmarinus officinalis* (Labiatae)	Antimicrobial activity; inhibition of mouse skin tumorigenesis; anti-inflammatory	[85, 119]

*Salvia miltiorrhiza* (Lamiaceae)	Inhibition of atherosclerosis	[120]

*Saprosma merrillii* (Rubiaceae)	Cytotoxic	[121]

*Siphonodon celastrineus* (Celastraceae)	Cytotoxic	[71]

*Solanum incanum* (Solanaceae)	Hepatoprotection	[122]

*Symplocos lancifolia* (Symplocaceae)	Antibacterial	[123]

*Teucrium viscidum* (Lamiaceae)	Inhibition of activities against 11*β*-HSD1	[124]

*Triplerospermum taiwanense* (Gentianaceae)	Hepatoprotection	[125]

*Uncaria laevigata* (Rubiaceae)	Inhibition of *α*-glucosidase	[81]

*Uncaria sessilifructus* (Rubiaceae)	Inhibition of activities against LPS-induced nitric oxide production in RAW264.7 macrophages	[82]

*Vladimiria muliensis* (Asteraceae)	Antimicrobial	[126]

*Weigela subsessilis* (Caprifoliaceae)	Diabetes treatment	[127]
	Anticomplementary	[128]

## References

[1] Oldfield E., Lin F.-Y. (2012). Terpene biosynthesis: modularity rules. *Angewandte Chemie International Edition*.

[2] Hill R. A., Connolly J. D. (2012). Triterpenoids. *Natural Product Reports*.

[3] Hill R. A., Connolly J. D. (2011). Triterpenoids. *Natural Product Reports*.

[4] Patlolla J. M. R., Rao C. V. (2012). Triterpenoids for cancer prevention and treatment: current status and future prospects. *Current Pharmaceutical Biotechnology*.

[5] Cárdenas C., Quesada A. R., Medina M. Á. (2004). Effects of ursolic acid on different steps of the angiogenic process. *Biochemical and Biophysical Research Communications*.

[6] Ovesná Z., Vachálková A., Horváthová K., Tóthová D. (2004). Pentacyclic triterpenoic acids: new chemoprotective compounds. Minireview. *Neoplasma*.

[7] Dorai T., Aggarwal B. B. (2004). Role of chemopreventive agents in cancer therapy. *Cancer Letters*.

[8] Zhang P., Li H., Chen D., Ni J., Kang Y., Wang S. (2007). Oleanolic acid induces apoptosis in human Leukemia cells through caspase activation and poly(ADP-ribose) polymerase cleavage. *Acta Biochimica et Biophysica Sinica*.

[9] Bonaccorsi I., Altieri F., Sciamanna I. (2008). Endogenous reverse transcriptase as a mediator of ursolic acid's anti-proliferative and differentiating effects in human cancer cell lines. *Cancer Letters*.

[10] Xavier C. P. R., Lima C. F., Preto A., Seruca R., Fernandes-Ferreira M., Pereira-Wilson C. (2009). Luteolin, quercetin and ursolic acid are potent inhibitors of proliferation and inducers of apoptosis in both KRAS and BRAF mutated human colorectal cancer cells. *Cancer Letters*.

[11] Petronellia A., Pannitterib G., Testaa U. (2009). Triterpenoids as new promising anticancer drugs. *Anti-Cancer Drugs*.

[12] Liu J. (1995). Pharmacology of oleanolic acid and ursolic acid. *Journal of Ethnopharmacology*.

[13] Xu K., Chu F., Li G. (2014). Oleanolic acid synthetic oligoglycosides: a review on recent progress in biological activities. *Pharmazie*.

[14] Fukushima E. O., Seki H., Ohyama K. (2011). CYP716A subfamily members are multifunctional oxidases in triterpenoid biosynthesis. *Plant and Cell Physiology*.

[15] Simonsen J. L., Ross W. C. J. (1957). Hydroxy acids, hydroxy lactones, hydroxyaldehydo acids, hydroxyketo acids and the stereochemistry of the triterpenes. *The Terpenes: The Triterpenes and Their Derivatives*.

[16] Sporn M. B., Liby K. T., Yore M. M., Fu L., Lopchuk J. M., Gribble G. W. (2011). New synthetic triterpenoids: potent agents for prevention and treatment of tissue injury caused by inflammatory and oxidative stress. *Journal of Natural Products*.

[17] Heinzen R. A., Scidmore M. A., Rockey D. D., Hackstadt T. (1996). Differential interaction with endocytic and exocytic pathways distinguish parasitophorous vacuoles of *Coxiella burnetii* and *Chlamydia trachomatis*. *Infection and Immunity*.

[18] Szakiel A., Grzelak A., Dudek P., Janiszowska W. (2003). Biosynthesis of oleanolic acid and its glycosides in *Calendula officinalis* suspension culture. *Plant Physiology and Biochemistry*.

[19] Van L. T. K., Hung T. M., Thuong P. T. (2009). Oleanane-type triterpenoids from *Aceriphyllum rossii* and their cytotoxic activity. *Journal of Natural Products*.

[20] Min B.-S., Lee I., Chang M.-J. (2008). Anticomplementary activity of triterpenoids from the whole plant of *Aceriphyllum rossii* against the classical pathway. *Planta Medica*.

[21] Min B. S. (2012). Anticomplementary activity of oleanane-type triterpenes from the roots of *Aceriphyllum rossii*. *Archives of Pharmacal Research*.

[22] Zhou X.-F., Zhang P., Pi H.-F. (2009). Triterpenoids from the roots of *Actinidia chinensis*. *Chemistry and Biodiversity*.

[23] Wang B., Jiang Z. H. (1992). Studies on oleanolic acid. *Chinese Pharmaceutical Journal*.

[24] Liu J., Liu Y. P., Klaassen C. D. (1994). The effect of Chinese hepatoprotective medicines on experimental liver injury in mice. *Journal of Ethnopharmacology*.

[25] Sun H.-X., Ye Y.-P., Pan Y.-J. (2004). Cytotoxic oleanane triterpenoids from the rhizomes of *Astilbe chinensis* (Maxim.) Franch. et Savat.. *Journal of Ethnopharmacology*.

[26] Passero L. F. D., Bonfim-Melo A., Corbett C. E. P. (2011). Anti-leishmanial effects of purified compounds from aerial parts of *Baccharis uncinella* CDC (Asteraceae). *Parasitology Research*.

[27] Yamamoto E. S., Campos B. L. S., Laurenti M. D. (2014). Treatment with triterpenic fraction purified from *Baccharis uncinella* leaves inhibits Leishmania (*Leishmania*) amazonensis spreading and improves Th1 immune response in infected mice. *Parasitology Research*.

[28] Deng J.-Z., Starck S. R., Hecht S. M. (1999). DNA polymerase *β* inhibitors from *Baeckea gunniana*. *Journal of Natural Products*.

[29] Yabuchi T., Tanaka T., Sasatsuka T., Yamahara J., Fujimura H. (1988). Extraction of oleanolic acid from sugar beets for treatment of liver failure. *Chemical Abstracts*.

[30] Liu J. (1995). Oleanolic acid and ursolic acid: research perspectives. *Journal of Ethnopharmacology*.

[31] Yamaguchi C., In Y., Wada S.-I., Yamada T., Tokuda H., Tanaka R. (2009). Cancer chemopreventive activity of oleanane-type triterpenoids from the stem bark of *Betula ermanii*. *Chemistry and Biodiversity*.

[32] Favel A., Steinmetz M. D., Regli P., Vidal-Ollivier E., Elias R., Balansard G. (1994). *In vitro* antifungal activity of triterpenoid saponins. *Planta Medica*.

[33] Lu M.-Y., Liao Z.-X., Ji L.-J., Sun H.-F. (2013). Triterpenoids of *Chrysosplenium carnosum*. *Fitoterapia*.

[34] Phuong T. T., Chul H. L., Trong T. D. (2008). Triterpenoids from the leaves of *Diospyros kaki* (Persimmon) and their inhibitory effects on protein tyrosine phosphatase 1B. *Journal of Natural Products*.

[35] He X.-F., Wang X.-N., Gan L.-S., Yin S., Dong L., Yue J.-M. (2008). Two novel triterpenoids from *Dysoxylum hainanense*. *Organic Letters*.

[36] Lee M. K., Yang H., Yoon J. S. (2008). Antifibrotic activity of diterpenes from *Biota orientalis* leaves on hepatic stellate cells. *Archives of Pharmacal Research*.

[37] Machocho A. K., Kiprono P. C., Grinberg S., Bittner S. (2003). Pentacyclic triterpenoids from *Embelia schimperi*. *Phytochemistry*.

[38] Balanehru S., Nagarajan B. (1991). Protective effect of oleanolic acid and ursolic acid against lipid peroxidation. *Biochemistry International*.

[39] Balanehru S., Nagarajan B. (1992). Intervention of adriamycin induced free radical damage. *Biochemistry International*.

[40] Rajasekaran M., Bapna J. S., Lakshmanan S., Nair A. G. R., Veliath A. J., Panchanadam M. (1988). Antifertility effect in male rats of oleanolic acid, a triterpene from *Eugenia jambolana* flowers. *Journal of Ethnopharmacology*.

[41] Lai Y.-C., Chen C.-K., Tsai S.-F., Lee S.-S. (2012). Triterpenes as *α*-glucosidase inhibitors from *Fagus hayatae*. *Phytochemistry*.

[42] Cheng S.-Y., Wang C.-M., Hsu Y.-M. (2011). Oleanane-type triterpenoids from the leaves and twigs of *Fatsia polycarpa*. *Journal of Natural Products*.

[43] Hada S., Hattori T., Namba T. (1990). Dental caries prevention by traditional medicines—effect of components of *Ganoderma lucidum* on ghrcosyltransferase from *Streptococcusmutans*. *Chemical Abstracts*.

[44] Yoshimi N., Wang A., Morishita Y. (1992). Modifying effects of fungal and herb metabolites on azoxymethane-induced intestinal carcinogenesis in rats. *Japanese Journal of Cancer Research*.

[45] Ohigashi H., Takamura H., Koshimizu K., Tokuda H., Ito Y. (1986). Search for possible antitumor promoters by inhibition of 12-O-tetradecanoylphorbol-13-acetate-induced Epstein-Barr virus activation; Ursolic acid and oleanolic acid from an anti-inflammatory Chinese medicinal plant, *Glechoma hederaceae* L.. *Cancer Letters*.

[46] Tokuda H., Ohigashi H., Koshimizu K., Ito Y. (1986). Inhibitory effects of ursolic and oleanolic ancid on skin tumor promotion by 12-*O*-tetradecanoylphorbol-13-acetate. *Cancer Letters*.

[47] Nishimura K., Fukuda T., Miyase T., Noguchi H., Chen X.-M. (1999). Activity-guided isolation of triterpenoid acyl CoA cholesteryl acyl transferase (ACAT) inhibitors from *Ilex kudincha*. *Journal of Natural Products*.

[48] Pungitore C. R., Padrón J. M., Leon L. G. (2007). Inhibition of DNA topoisomerase I and growth inhibition of human cancer cell lines by an oleanane from *Junellia aspera* (Verbenaceae). *Cellular and Molecular Biology*.

[49] Jiménez-Arellanes A., Meckes M., Torres J., Luna-Herrera J. (2007). Antimycobacterial triterpenoids from *Lantana hispida* (Verbenaceae). *Journal of Ethnopharmacology*.

[50] Dat N. T., van Kiem P., Cai X. F., Shen Q., Bae K., Kim Y. H. (2004). Gymnastone, a new benzofuran derivative from *Gymnaster koraiensis*. *Archives of Pharmacal Research*.

[51] Dai Y., Hang B.-Q., Meng Q.-Y., Ma S.-P., Tan L.-W. (1988). Inhibition of hypersensitivity reactions by oleanolic acid. *Zhongguo Yao Li Xue Bao*.

[52] Dai Y., Hang B. Q., Li P. Z., Tan L. W. (1989). Effects of oleanolic acid on immune system and type I allergic reaction. *Zhongguo Yao Li Xue Bao*.

[53] Liu J., Liu Y., Mao Q., Klaassen C. D. (1994). The effects of 10 triterpenoid compounds on experimental liver injury in mice. *Fundamental and Applied Toxicology*.

[54] Niikawa M., Hayashi H., Sato T., Nagase H., Kito H. (1993). Isolation of substances from glossy privet (*Ligustrum lucidum Ait*.) inhibiting the mutagenicity of benzo[a]pyrene in bacteria. *Mutation Research*.

[55] Singh G. B., Singh S., Bani S., Gupta B. D., Banerjee S. K. (1992). Anti-inflammatory activity of oleanolic acid in rats and mice. *Journal of Pharmacy and Pharmacology*.

[56] Kapil A., Sharma S. (1994). Anti-complement activity of oleanolic acid: an inhibitor of C3-convertase of the classical complement pathway. *Journal of Pharmacy and Pharmacology*.

[57] Huang X.-A., Shen X.-L., Hu Y.-J. (2011). Two new triterpenoids from *Lysimachia heterogenea* klatt and evaluation of their cytotoxicity. *Molecules*.

[58] He Z., Liang F., Lu J., Pan Y. (2013). Cytotoxic triterpenoids from *Lysimachia parvifolia*. *European Journal of Medicinal Chemistry*.

[59] Sánchez M., Mazzuca M., Veloso M. J. (2010). Cytotoxic terpenoids from *Nardophyllum bryoides*. *Phytochemistry*.

[60] Chen I.-H., Lu M.-C., Du Y.-C. (2009). Cytotoxic triterpenoids from the stems of *Microtropis japonica*. *Journal of Natural Products*.

[61] Tian Z., Liu Y.-M., Chen S.-B. (2006). Cytotoxicity of two triterpenoids from *Nigella glandulifera*. *Molecules*.

[62] Gupta M. B., Bhalla T. N., Gupta G. P., Mitra C. R., Bhargava K. P. (1969). Anti-inflammatory activity of natural products (I) Triterpenoids. *European Journal of Pharmacology*.

[63] Shibata S., Wagner H., Wolff P. (1977). Saponins with biological and pharmacological activity. *New Natural Products And Plant Drugs with Pharmacologicalor Therapeutical Activity*.

[64] Liang C., Ding Y., Nguyen H. T. (2010). Oleanane-type triterpenoids from *Panax stipuleanatus* and their anticancer activities. *Bioorganic & Medicinal Chemistry Letters*.

[65] Liang C., Ding Y., Song S. B. (2013). Oleanane-triterpenoids from *Panax stipuleanatus* inhibit NF-*κ*B. *Journal of Ginseng Research*.

[66] Wada S.-I., Iida A., Tanaka R. (2001). Screening of triterpenoids isolated from *Phyllanthus flexuosus* for DNA topoisomerase inhibitory activity. *Journal of Natural Products*.

[67] Zhan Q., Zhang F., Sun L., Wu Z., Chen W. (2012). Two new oleanane-type triterpenoids from platycodi radix and anti-proliferative activity in HSC-T6 Cells. *Molecules*.

[68] Zeng N., Shen Y., Li L.-Z. (2011). Anti-inflammatory triterpenes from the leaves of *Rosa laevigata*. *Journal of Natural Products*.

[69] Yan M., Zhu Y., Zhang H.-J. (2013). Anti-inflammatory secondary metabolites from the leaves of *Rosa laevigata*. *Bioorganic & Medicinal Chemistry*.

[70] Takagi K., Park E. K., Kato H. (1980). Anti-inflammatory activities of hederagenin and crude saponin isolated from *Sapindus mukorossi* GAERTN. *Chemical and Pharmaceutical Bulletin*.

[71] Kaweetripob W., Mahidol C., Prawat H., Ruchirawat S. (2013). Lupane, friedelane, oleanane, and ursane triterpenes from the stem of *Siphonodon celastrineus* Griff. *Phytochemistry*.

[72] Niampoka C., Suttisri R., Bavovada R., Takayama H., Aimi N. (2005). Potentially cytotoxic triterpenoids from the root bark of *Siphonodon celastrineus* Griff. *Archives of Pharmacal Research*.

[73] Hunan Med Inst (1975). Pharmacological studied of hepatoprotective compounds from *Swertia mileensis*. *Traditional Medicine (Zhong Chao Yao)*.

[74] Human Medicine Institute (1977). Effects of oleanolic acid on experimental liver injury and therapeutic value in human hepatitis. *Traditional Medicine*.

[75] Ma X. H., Zhao Y. C., Yin L., Han D. W., Ji C. X. (1982). Studies on the effect of oleanolic acid on experimental liver injury. *Yao Xue Xue Bao*.

[76] Hikino H., Ohsawa T., Kiso Y., Oshima Y. (1984). Analgesic and antihepatotoxic actions of dianosides, triterpenoid saponins of *Dianthus superbus* var. *longicalycinus* Herbs. *Planta Medica*.

[77] Pawar R. S., Bhutani K. K. (2005). Effect of oleanane triterpenoids from *Terminalia arjuna*—a cardioprotective drug on the process of respiratory oxyburst. *Phytomedicine*.

[78] Manosroi A., Jantrawut P., Akazawa H., Akihisa T., Manosroi J. (2010). Biological activities of phenolic compounds isolated from galls of *Terminalia chebula* Retz. (Combretaceae). *Natural Product Research*.

[79] Hiroshi H., Yoshinobu K., Sakae A., Yukio O. (1984). Antihepatotoxic actions of papyriogenins and papyriosides, triterpenoids of *Tetrapanax papyriferum* leaves. *Journal of Ethnopharmacology*.

[80] Hao Z., Hang B., Wang Y. (1989). Hypoglycemic effect of oleanolic acid. *Zhougguo Yaoke Daxue Xuebao*.

[81] Wang Z.-W., Wang J.-S., Luo J., Kong L.-Y. (2013). *α*-glucosidase inhibitory triterpenoids from the stem barks of *Uncaria laevigata*. *Fitoterapia*.

[82] Zhang M.-J., Liu B., Liao S.-G. (2013). Uncarilic acid and secouncarilic acid, two new triterpenoids from *Uucaria sessilifructus*. *Molecules*.

[83] Chen X.-Q., Li Y., He J. (2011). Triterpenoids and diterpenoids from *Viburnum chingii*. *Chemical and Pharmaceutical Bulletin*.

[84] Huang M.-T., Ho C.-T., Wang Z. Y. (1994). Inhibition of skin tumorigenesis by rosemary and its constituents carnosol and ursolic acid. *Cancer Research*.

[85] Kondo M. (2006). *Phytochemical studies of extracts from cranberry (Vaccinium macrocarpon) with anti-cancer, anti-fungal and cardioprotective properties [M.S. thesis]*.

[86] Varanda E. M., Zúñiga G. E., Salatino A., Roque N. F., Corcuera L. J. (1992). Effect of ursolic acid from epicuticular waxes of *Jacaranda decurrens* on *Schizaphis graminum*. *Journal of Natural Products*.

[87] Connolly J. D., Hill R. A. (1999). Triterpenoids. *Natural Product Reports*.

[88] Safayhi H., Sailer E.-R. (1997). Anti-inflammatory actions of pentacyclic triterpenes. *Planta Medica*.

[89] Ríos J.-L. (2010). Effects of triterpenes on the immune system. *Journal of Ethnopharmacology*.

[90] Baglin I., Poumaroux A., Nour M. (2003). New ursolic and betulinic derivatives as potential cytotoxic agents. *Journal of Enzyme Inhibition and Medicinal Chemistry*.

[91] Chinou I., Liolios C. H., Moreau D., Roussakis C. H. (2007). Cytotoxic activity of *Origanum dictamnus*. *Fitoterapia*.

[92] Cunha W. R., Martins C., Ferreira D. D. S., Crotti A. E. M., Lopes N. P., Albuquerque S. (2003). *In Vitro* trypanocidal activity of triterpenes from *Miconia* species. *Planta Medica*.

[93] Taketa A. T. C., Gnoatto S. C. B., Gosmann G., Pires V. S., Schenkel E. P., Guillaume D. (2004). Triterpenoids from Brazilian *Ilex* species and their in vitro antitrypanosomal activity. *Journal of Natural Products*.

[94] Traore-Keita F., Gasquet M., di Giorgio C. (2000). Antimalarial activity of four plants used in traditional medicine in Mali. *Phytotherapy Research*.

[95] Torres-Santos E. C., Lopes D., Oliveira R. R. (2004). Antileishmanial activity of isolated triterpenoids from *Pourouma guianensis*. *Phytomedicine*.

[96] Kozai K., Miyake Y., Kohda H. (1987). Inhibition of glucosyltransferase from *Streptococcus mutans* by oleanolic acid and ursolic acid. *Caries Research*.

[97] Kim M. J., Kim C. S., Park J. Y. (2011). Antimicrobial effects of ursolic acid against mutans Streptococci isolated from Koreans. *International Journal of Oral Science*.

[98] Jiménez A., Meckes M., Alvarez V., Torres J., Parra R. (2005). Secondary metabolites from *Chamaedora tepejilote* (Palmae) are active against *Mycobacterium tuberculosis*. *Phytotherapy Research*.

[99] Zhou X.-F., Zhang P., Pi H.-F. (2009). Triterpenoids from the roots of *Actinidia chinensis*. *Chemistry & Biodiversity*.

[100] Simon A., Najid A., Chulia A. J., Delage C., Rigaud M. (1992). Inhibition of lipoxygenase activity and HL60 leukemic cell proliferation by ursolic acid isolated from heather flowers (*Calluna vulgaris*). *Biochimica et Biophysica Acta (BBA)—Lipids and Lipid Metabolism*.

[101] Najid A., Simon A., Cook J. (1992). Characterization of ursolic acid as a lipoxygenase and cyclooxygenase inhibitor using macrophages, platelets and differentiated HL60 leukemic cells. *FEBS Letters*.

[102] Nhiem N. X., Tai B. H., Quang T. H. (2011). A new ursane-type triterpenoid glycoside from Centella asiatica leaves modulates the production of nitric oxide and secretion of TNF-*α* in activated RAW 264.7 cells. *Bioorganic & Medicinal Chemistry Letters*.

[103] Wu X.-D., He J., Li X.-Y. (2013). Triterpenoids and steroids with cytotoxic activity from *Emmenopterys henryi*. *Planta Medica*.

[104] Young H.-S., Chung H.-Y., Lee C.-K., Park K.-Y., Yokozawa T., Oura H. (1994). Ursolic acid inhibits aflatoxin B_1_-induced mutagenicity in a Salmonella assay system. *Biological and Pharmaceutical Bulletin*.

[105] Shukla B., Visen P. K. S., Patnaik G. K. (1992). Hepatoprotective activity in the rat of ursolic acid isolated from Eucalyptus hybrid. *Phytotherapy Research*.

[106] Sidana J., Singh S., Arora S. K., Foley W. J., Singh I. P. (2012). Terpenoidal constituents of *Eucalyptus loxophleba ssp. lissophloia*. *Pharmaceutical Biology*.

[107] Song N.-Y., Cho J.-G., Im D. (2013). Triterpenoids from *Fragaria ananassa* calyx and their inhibitory effects on melanogenesis in B16-F10 mouse melanoma cells. *Natural Product Research: Formerly Natural Product Letters*.

[108] Wu Q.-X., Liu X., Shi Y.-P. (2007). Chemical components from *Gentiana aristata*. *Chemistry & Biodiversity*.

[109] Wang W.-L., Zhou X., Liu Y.-L., Xu Q.-M., Li X.-R., Yang S.-L. (2014). Two new 20*α*(H)-ursane-type triterpenoids from *Ilex cornuta* cornuta and their cytotoxic activities. *Journal of Asian Natural Products Research*.

[110] Ali M. S., Ibrahim S. A., Jalil S., Choudhary M. I. (2007). Ursolic acid: a potent Inhibitor of superoxides produced in the cellular system. *Phytotherapy Research*.

[111] Tsuruga T., Chun Y.-T., Ebizuka Y., Sankawa U. (1991). Biologically active constituents of *Melaleuca leucadendron*: inhibitors of induced histamine release from rat mast cells. *Chemical and Pharmaceutical Bulletin*.

[112] Wang X.-X., Lin C.-J., Jia Z.-J. (2006). Triterpenoids and sesquiterpenes from *Mulgedium tataricum*. *Planta Medica*.

[113] Tao J.-Y., Dai S.-J., Zhao F., Liu J.-F., Fang W.-S., Liu K. (2012). New ursane-type triterpene with NO production suppressing activity from *Nauclea officinalis*. *Journal of Asian Natural Products Research*.

[114] Xie W.-D., Zhang Q., Li P.-L., Jia Z.-J. (2005). Two triterpenoids and other constituents from *Petasites tricholobus*. *Phytochemistry*.

[115] Choudhary A., Mittal A. K., Radhika M. (2013). Two new stereoisomeric antioxidant triterpenes from *Potentilla fulgens*. *Fitoterapia*.

[116] Kosuge T., Yokota M., Sugiyama K., Mure T., Yamazawa H., Yamamoto T. (1985). Studies on bioactive substances in crude drugs used for arthritic diseases in traditional Chinese medicine. III. Isolation and identification of anti-inflammatory and analgesic principles from the whole herb of *Pyrola rotundifolia* L. *Chemical and Pharmaceutical Bulletin*.

[117] Lee K.-H., Lin Y.-M., Wu T.-S. (1988). The cytotoxic principles of *Prunella vulgaris*, *Psychotria serpens*, and *Hyptis capitata*: ursolic acid and related derivatives. *Planta Medica*.

[118] Choi Y. H., Zhou W., Oh J. (2012). Rhododendric acid A, a new ursane-type PTP1B inhibitor from the endangered plant *Rhododendron brachycarpum* G. Don. *Bioorganic and Medicinal Chemistry Letters*.

[119] Collins M. A., Charles H. P. (1987). Antimicrobial activity of Carnosol and Ursolic acid: two anti-oxidant constituents of *Rosmarinus officinalis* L.. *Food Microbiology*.

[120] Steinkamp-Fenske K., Bollinger L., Völler N. (2007). Ursolic acid from the Chinese herb danshen (*Salvia miltiorrhiza* L.) upregulates eNOS and downregulates Nox4 expression in human endothelial cells. *Atherosclerosis*.

[121] Zhang D., Chen W., Song X., Han C., Wang Y., Chen G. (2013). Three new ursane-type triterpenoids from the stems of *Saprosma merrillii*. *Molecules*.

[122] Lin C.-N., Chung M.-I., Gan K.-H. (1988). Novel antihepatotoxic principles of *Solanum incanum*. *Planta Medica*.

[123] Acebey-Castellon I. L., Voutquenne-Nazabadioko L., Mai H. D. T. (2011). Triterpenoid saponins from *Symplocos lancifolia*. *Journal of Natural Products*.

[124] Hao X., Zhang J., Xia G. (2013). A new triterpenoid from *Teucrium viscidum*. *Molecules*.

[125] Gan K. H., Lin C. N. (1988). Studies on the constituents of Fonnosan gentianaceous plants XI—*Constituents of Gentiana flavo-maculata* and *Tripterospennum taiwanense* and the antihepatotoxic activity of ursolic acid derivatives. *Chinese Pharmceutical Journal*.

[126] Chen J.-J., Fei D.-Q., Chen S.-G., Gao K. (2008). Antimicrobial triterpenoids from *Vladimiria muliensis*. *Journal of Natural Products*.

[127] Lee J., Yee S.-T., Kim J.-J. (2010). Ursolic acid ameliorates thymic atrophy and hyperglycemia in streptozotocin-nicotinamide-induced diabetic mice. *Chemico-Biological Interactions*.

[128] Thuong P. T., Min B.-S., Jin W. (2006). Anti-complementary activity of ursane-type triterpenoids from *Weigela subsessilis*. *Biological and Pharmaceutical Bulletin*.

[129] Woldemichael G. M., Franzblau S. G., Zhang F., Wang Y., Timmermann B. N. (2003). Inhibitory effect of sterols from *Ruprechtia triflora* and diterpenes from *Calceolaria pinnifolia* on the growth of *Mycobacterium tuberculosis*. *Planta Medica*.

[130] Horiuchi K., Shiota S., Hatano T., Yoshida T., Kuroda T., Tsuchiya T. (2007). Antimicrobial activity of oleanolic acid from *Salvia officinalis* and related compounds on vancomycin-resistant enterococci (VRE). *Biological & Pharmaceutical Bulletin*.

[131] Fontanay S., Grare M., Mayer J., Finance C., Duval R. E. (2008). Ursolic, oleanolic and betulinic acids: antibacterial spectra and selectivity indexes. *Journal of Ethnopharmacology*.

[132] Cunha W. R., de Matos G. X., Souza M. G. M. (2010). Evaluation of the antibacterial activity of the methylene chloride extract of *Miconia ligustroides*, isolated triterpene acids, and ursolic acid derivatives. *Pharmaceutical Biology*.

[133] Martins A., Vasas A., Viveiros M., Molnár J., Hohmann J., Amaral L. (2011). Antibacterial properties of compounds isolated from *Carpobrotus edulis*. *International Journal of Antimicrobial Agents*.

[134] Grudniak A. M., Kurek A., Szarlak J., Wolska K. I. (2011). Oleanolic and ursolic acids influence affect the expression of the cysteine regulon and the stress response in Escherichia coli. *Current Microbiology*.

[135] Kurek A., Grudniak A. M., Szwed M. (2010). Oleanolic acid and ursolic acid affect peptidoglycan metabolism in *Listeria monocytogenes*. *Antonie van Leeuwenhoek*.

[136] Girard M. P., Osmanov S., Assossou O. M., Kieny M.-P. (2011). Human immunodeficiency virus (HIV) immunopathogenesis and vaccine development: a review. *Vaccine*.

[137] Ma C., Nakamura N., Miyashiro H., Hattori M., Shimotohno K. (1999). Inhibitory effects of constituents from *Cynomorium songaricum* and related triterpene derivatives on HIV-1 protease. *Chemical and Pharmaceutical Bulletin*.

[138] Nakamura N. (2004). Inhibitory effects of some traditional medicines on proliferation of HIV-1 and its protease. *Yakugaku Zasshi*.

[139] Filho J. R., Falcão H. D. S., Batista L. M., Filho J. M. B., Piuvezam M. R. (2010). Effects of plant extracts on HIV-1 protease. *Current HIV Research*.

[140] Mengoni F., Lichtner M., Battinelli L. (2002). *In vitro* anti-HIV activity of oleanolic acid on infected human mononuclear cells. *Planta Medica*.

[141] Kashiwada Y., Wang H. K., Nagao T. (1998). Anti-AIDS agents—anti-HIV activity of pomolic and structurally related triterpenoids. *Journal Natural Products*.

[142] Hattori M., Ma C. M., Wei Y., Dine S. R. E., Sato N. (2013). Survey of anti-HIV and anti-HCV compounds from *Natural sources*. *Canadian Chemical Transactions*.

[143] Shyu M.-H., Kao T.-C., Yen G.-C. (2010). Oleanolic acid and ursolic acid induce apoptosis in HuH7 human hepatocellular carcinoma cells through a mitochondrial-dependent pathway and downregulation of XIAP. *Journal of Agricultural and Food Chemistry*.

[144] Wu H.-Y., Chang C.-I., Lin B.-W. (2011). Suppression of hepatitis B virus X protein-mediated tumorigenic effects by ursolic acid. *Journal of Agricultural and Food Chemistry*.

[145] Kong L., Li S., Liao Q. (2013). Oleanolic acid and ursolic acid: novel hepatitis C virus antivirals that inhibit NS5B activity. *Antiviral Research*.

[146] Bag P., Chattopadhyay D., Mukherjee H. (2012). Anti-herpes virus activities of bioactive fraction and isolated pure constituent of *Mallotus peltatus*: an ethnomedicine from Andaman Islands. *Virology Journal*.

[147] Bhargava N. (1983). Ethnobotanical studies of the tribes of Andaman and Nicobar Islands, India. I. Onge. *Economic Botany*.

[148] Goyal B. R., Goyal R. K., Mehta A. A. (2007). Phyto-pharmacology of *Achyranthes aspera*: a review. *Pharmacognosy Reviews*.

[149] Chen Y., Zhu Z., Guo Q., Zhang L., Zhang X. (2012). Variation in concentrations of major bioactive compounds in *Prunella vulgaris* L. related to plant parts and phenological stages. *Biological Research*.

[150] Mukherjee H., Ojha D., Bag P. (2013). Anti-herpes virus activities of *Achyranthes aspera*: an Indian ethnomedicine, and its triterpene acid. *Microbiological Research*.

[151] Nishikomori R., Gurunathan S., Nishikomori K., Strober W. (2001). BALB/c mice bearing a transgenic IL-12 receptor *β*2 gene exhibit a nonhealing phenotype to *Leishmania major* infection despite intact IL-12 signaling. *The Journal of Immunology*.

[152] Passero L. F. D., Bordon M. L. A. D. C., de Carvalho A. K., Martins L. M., Corbett C. E. P., Laurenti M. D. (2010). Exacerbation of *Leishmania (Viannia) shawi* infection in BALB/c mice after immunization with soluble antigen from amastigote forms. *APMIS*.

[153] Oliveira-Ferreira J., Lacerda M. V. G., Brasil P., Ladislau J. L. B., Tauil P. L., Daniel-Ribeiro C. T. (2010). Malaria in Brazil: an overview. *Malaria Journal*.

[154] Sibley C. H., Price R. N. (2012). Monitoring antimalarial drug resistance: applying lessons learned from the past in a fast-moving present. *International Journal for Parasitology: Drugs and Drug Resistance*.

[155] Steele J. C. P., Warhurst D. C., Kirby G. C., Simmonds M. S. J. (1999). *In vitro* and *in vivo* evaluation of betulinic acid as an antimalarial. *Phytotherapy Research*.

[156] van Baren C., Anao I., Lira P. D. L. (2006). Triterpenic acids and flavonoids from *Satureja parvifolia*, evaluation of their antiprotozoal activity. *Journal of Biosciences*.

[157] Simelane M. B. C., Shonhai A., Shode F. O., Smith P., Singh M., Opoku A. R. (2013). Anti-plasmodial activity of some zulu medicinal plants and of some triterpenes isolated from them. *Molecules*.

[158] Al Musayeib N. M., Mothana R. A., El Gamal A. A., Al-Massarani S. M., Maes L. (2013). *In Vitro* antiprotozoal activity of triterpenoid constituents of *Kleinia odora* growing in Saudi Arabia. *Molecules*.

[159] McGwire B. S., Satoskar A. R. (2013). Leishmaniasis: clinical syndromes and treatment. *Quarterly Journal of Medicine*.

[160] Tan N., Kaloga M., Radtke O. A. (2002). Abietane diterpenoids and triterpenoic acids from *Salvia cilicica* and their antileishmanial activities. *Phytochemistry*.

[161] Gnoatto S. C. B., Vechia L. D., Lencina C. L. (2008). Synthesis and preliminary evaluation of new ursolic and oleanolic acids derivatives as antileishmanial agents. *Journal of Enzyme Inhibition and Medicinal Chemistry*.

[162] Filho A. A. D. S., Resende D. O., Fukui M. J. (2009). *In vitro* antileishmanial, antiplasmodial and cytotoxic activities of phenolics and triterpenoids from *Baccharis dracunculifolia* D. C. (Asteraceae). *Fitoterapia*.

[163] Tarleton R. L. (2003). *Trypanosoma cruzi* and chagas disease: cause and effect. *American Trypanosomiasis*.

[164] Stahl P., Ruppert V., Schwarz R. T., Meyer T. (2014). *Trypanosoma cruzi* evades the protective role of interferon-gamma-signaling in parasite-infected cells. *PLoS ONE*.

[165] Coura J. R., de Castro S. L. (2002). A critical review on chagas disease chemotherapy. *Memorias do Instituto Oswaldo Cruz*.

[166] Cunha W. R., Crevelin E. J., Arantes G. M. (2006). A study of the trypanocidal activity of triterpene acids isolated from *Miconia* species. *Phytotherapy Research*.

[167] Ferreira D. D. F., Esperandim V. R., Toldo M. P. A., Saraiva J., Cunha W. R., de Albuquerque S. (2010). Trypanocidal activity and acute toxicity assessment of triterpene acids. *Parasitology Research*.

